# Supplementary material for: Association between Interferon-Lambda-3 rs12979860, TLL1 rs17047200 and DDR1 rs4618569 Variant Polymorphisms with the Course and Outcome of SARS-CoV-2 Patients
Source: Genes (Basel). 2021 May 28;12(6):830. doi: 10.3390/genes12060830 (PMC8230293; doi:10.3390/genes12060830)
Supplement: Supplementary file 1 [file genes-12-00830-s001.zip › genes-1231606-supplementary.pdf]

Supplementary Table 1. Demographic and laboratory characters of Covid-19 cases according to genotype distribution:

	SNP1 (rs12979860)			SNP 2 (rs17047200)			SNP 3 (rs4618569)			Test of significance
	**TT**<br>***n* = 19**	**TC**<br>***n* = 59**	**CC**<br>***n* = 63**	**AA**<br>***n* = 94**	**TA**<br>***n* = 37**	**TT**<br>***n* = 10**	**AA**<br>***n* = 66**	**AG**<br>***n* = 56**	**GG**<br>***n* = 19**	
Sex<br>Male 85(60.3%)<br>Female 56<br>(39.7%)	12(63.2%)<br>7(36.8%)	36(61%)<br>23(39%)	37(58.7%)<br>26(41.3%)	55(58.5%)<br>39 (41.5%)	22(59.5%)<br>15(%)	8(80%)<br>2(20%)	44(66.7%)<br>22 (33.3%)	29(51.8%)<br>27 (48.2%)	12(63.2%)<br>7 (36.8%)	X^2=.142,<br>P1=.931<br>X^2=1.758,<br>P2=.415<br>X^2=2.878,<br>P3=.237
Comorbidities<br>+ve<br>81(57.4%)<br>-ve 60(42.6%)	14(73.7%)<br>5(26.3%)	31(52.5%)<br>28(47.5%)	36(57.1%)<br>27(42.9%)	54(57.4%)<br>40(42.6%)	20(54.1%)<br>17(45.9%)	7(70%)<br>3(30%)	31(47%)<br>35 (53%)	37(66.1%)<br>19(33.9%)	13(68.4%)<br>6(31.6%)	X^2=2.632,<br>P1=.268<br>X^2=.819,<br>P2=.664<br>X^2=5.604,<br>P3=.061
Severity<br>**Mild(*n* = 82)**<br>**Severe(*n* = 11)**<br>**Critical(*n* = 84)**	9 (47.4%)<br>0(0%)<br>9 (10.7%)	32 (54.2%)<br>3 (27.2%)<br>32 (38%)	41 (65.1%)<br>8 (72.8%)<br>43 (57.1%)	58 (61.7%)<br>0 (0%)<br>60 (71.4%	21 (56.8%)<br>2(9.2%<br>21 (25%)	3(30%)<br>0(0%)<br>7 (8.3%)<br>0	46(69.7%)<br>9(81.8%)<br>48(57.1%)	22(39.3%)<br>2(17.8%)<br>34(40.5%)	14(73.7%)<br>0(0%)<br>5(5.09%)	X^2=2.522,<br>P1=.283<br>X^2=3.773,<br>P2=.152<br>X^2=13.689,

										P3=.001*
Ventilation<br>+ve **54 (38.3%)**<br>**-ve 87 (61.7%)**	9 (47.4%)<br>10(52.6 %)	24 (40.7%)<br>35 (59.3%)	21 (33.3%)<br>42(66.7%)	31 (33%)<br>63 (67%)	16 (43.2%)<br>21 (56.8%)	7 (70%)<br>3 (30%)	17(25.8 %)<br>49(74.2 %)	32 (57.1%)<br>24(42.9 %)	5 (26.3%)<br>14 (73.7%)	X^2=1.460, P1=.482<br>X^2=5.761, P2=.056<br>X2=13.963, P3=.001*
Outcome<br>Recovery **63 (44.7%)**<br>Recurrence **30 (21.3%)**<br>Death **48 (34%)**	5(26.3 %)<br>6(31.6 %)<br>8(42.1 %)	27(45.8 %)<br>9(15.3%)<br>23(39%)	31(49.2%)<br>15(23.8%)<br>17(27%)	47 (50%)<br>18(19.1%)<br>29(30.9%)	13(35.1%)<br>10(27%)<br>14(37.8%)	3(30%)<br>2(20%)<br>5(50%)	33(50%)<br>17(25.8.3%)<br>16(24.2 %)	20(35.7 %)<br>7 (12.5%)<br>29(51.8 %)	10(52.6 %)<br>6(31.6 %)<br>3(15.8 %)	X^2=5.590, P1=.232<br>X^2=3.801, P2=.434<br>X^2=14.193, P3=.007*
**Age/years**	35±14.8	37.5±21.2	38±21.5	35.2±21.9	41.5±18.2	42.9±9.7	39.1±20.5	35.5±20.5	36.9±20.8	F1=.159, P1=.853<br>F2=1.640, P2=.198<br>F3=.457, P3=.634

**TLC (thousands/cmm)**	72.16	69.64	71.92	66.7	79.59	79.65	67.65	74.79	71.47	Kw1=.113, P1=.945 Kw2=3.143, P2=.208 Kw3=.931, P3=.628
**Ferritin(ng/mL)**	72.74	68.78	72.56	70.74	67.70	85.60	69.38	72.58	71.97	Kw1=.301, P1=.860 Kw2=1.526, P2=.466 Kw3=.199, P3=.905
**Lymphocytes (x10^9/L)**	61.71	74.47	70.55	76.01	60.07	64.40	72.42	70.50	67.55	Kw1=1.429, P1=.490 Kw2=4.357, P2=.113 Kw3=.255, P3=.894
**Haemoglobin (g/dL)**	12.4±2.2	12.2±2.1	11.8±1.9	12.2±2.05	11.7±2.07	12.1±2.3	12.05±2.05	11.99±2.16	12.54±1.91	F1=.776, P1=.462 F2=.668, P2=.514 F3=.535, P3=.587

**PLTs (thousands/cmm)**	269.3±55.5	256.2±67.5	261.3±92.1	260.4±86.7	249.6±55.9	299.1±43.4	258.25±77.7	266.5±75.6	249±87	F1=.209, P1=.812 F2=1.604, P2=.205 F3=401, P3=.671
**C-reactive protein (mg/L)**	68.13	66.81	75.79	64.23	82.77	91.1	69.84	72.46	70.71	Kw1=,1.582 P1=.453 Kw2=8.097, P2=.017* Kw3=.126, P3=.939
**Serum LDH (U/L)**	73.13	65.55	75.46	67.51	78.42	76.40	75.95	64.05	74.29	Kw1=1.859, P1=.395 Kw2=2.090, P2=.352 Kw3=2.719, P3=.257
**D-dimer(mg/L)**	75.34	71.31	69.40	69.85	76.23	69.06	68.41	74.35	70.13	Kw1=.319, P1=.853 Kw2=.838, P2=.658 Kw3=.660, P3=.719

P1: difference between TT, TC&CC, P2: difference between TT, AT&AA, P3: difference between AA, AG&GG KWχ2: Kruskal Wallis test, F: One Way ANOVA test, χ2: Chi-Square test, Parameters described as mean± SD for parametric values & mean rank for non-parametric values. *statistically significant

Supplementary Table 2. Correlation between the frequencies of polymorphisms, severity and mortality rates of Covid-19 cases (Spearman's rho):

		rs12979860_TT	rs12979860_TC	rs12979860_CC	rs17047200_AA	rs17047200_AT	rs17047200_TT	rs4618569_AA	rs4618569_AG	rs4618569_GG	Severity	Outcome
rs12979860_TT	Correlation Coefficient	1.000	-.335**	-.355**	-.117	.095	.053	-.120	.147	-.034	.086	.122
	Sig. (2-tailed)	.	.000	.000	.165	.262	.534	.155	.083	.688	.309	.149
	N	141	141	141	141	141	141	141	141	141	141	141
rs12979860_TC	Correlation Coefficient	-.335**	1.000	-.762**	.020	-.048	.046	.097	-.189*	.128	.067	.032
	Sig. (2-tailed)	.000	.	.000	.811	.568	.591	.250	.025	.129	.427	.707
	N	141	141	141	141	141	141	141	141	141	141	141
rs12979860_CC	Correlation Coefficient	-.355**	-.762**	1.000	.061	-.017	-.082	-.014	.087	-.104	-.126	-.116
	Sig. (2-tailed)	.000	.000	.	.476	.839	.336	.869	.306	.220	.136	.173
	N	141	141	141	141	141	141	141	141	141	141	141
rs17047200_AA	Correlation Coefficient	-.117	.020	.061	1.000	-.844**	-.391**	.060	-.041	-.029	-.102	-.139
	Sig. (2-tailed)	.165	.811	.476	.	.000	.000	.478	.629	.730	.230	.100
	N	141	141	141	141	141	141	141	141	141	141	141
rs17047200_AT	Correlation Coefficient	.095	-.048	-.017	-.844**	1.000	-.165	-.043	.010	.048	.017	.093
	Sig. (2-tailed)	.262	.568	.839	.000	.	.051	.616	.906	.573	.842	.270
	N	141	141	141	141	141	141	141	141	141	141	141

	rs17047200_TT	Correlation Coefficient	.053	.046	-.082	-.391**	-.165	1.000	-.038	.058	-.028	.158	.095
		Sig. (2-tailed)	.534	.591	.336	.000	.051	.	.657	.494	.741	.062	.260
		N	141	141	141	141	141	141	141	141	141	141	141
	rs4618569_AA	Correlation Coefficient	-.120	.097	-.014	.060	-.043	-.038	1.000	-.761**	-.370**	-.219**	-.156
		Sig. (2-tailed)	.155	.250	.869	.478	.616	.657	.	.000	.000	.009	.064
		N	141	141	141	141	141	141	141	141	141	141	141
	rs4618569_AG	Correlation Coefficient	.147	-.189*	.087	-.041	.010	.058	-.761**	1.000	-.320**	.310**	.238**
		Sig. (2-tailed)	.083	.025	.306	.629	.906	.494	.000	.	.000	.000	.004
		N	141	141	141	141	141	141	141	141	141	141	141
	rs4618569_GG	Correlation Coefficient	-.034	.128	-.104	-.029	.048	-.028	-.370**	-.320**	1.000	-.124	-.113
		Sig. (2-tailed)	.688	.129	.220	.730	.573	.741	.000	.000	.	.142	.182
		N	141	141	141	141	141	141	141	141	141	141	141
	Severity	Correlation Coefficient	.086	.067	-.126	-.102	.017	.158	-.219**	.310**	-.124	1.000	.828**
		Sig. (2-tailed)	.309	.427	.136	.230	.842	.062	.009	.000	.142	.	.000
		N	141	141	141	141	141	141	141	141	141	141	141
	Outcome	Correlation Coefficient	.122	.032	-.116	-.139	.093	.095	-.156	.238**	-.113	.828**	1.000
		Sig. (2-tailed)	.149	.707	.173	.100	.270	.260	.064	.004	.182	.000	.
		N	141	141	141	141	141	141	141	141	141	141	141

**. Correlation is significant at the 0.01 level (2-tailed).

*. Correlation is significant at the 0.05 level (2-tailed).
